# Phase II study of viscum fraxini-2 in patients with advanced hepatocellular carcinoma

**DOI:** 10.1038/sj.bjc.6601463

**Published:** 2004-01-06

**Authors:** M Mabed, L El-Helw, S Shamaa

**Affiliations:** 1 Hematology and Medical Oncology Unit, Faculty of Medicine, Mansoura University, Mansoura, Egypt

**Keywords:** chemotherapy, hepatocellular carcinoma, phase II, viscum fraxini-2

## Abstract

Hepatocellular carcinoma (HCC) is one of the most common cancers worldwide. Although a wide range of therapeutic options is available, the efficacy of these methods and the prognosis of patients with HCC remain very poor. This study was conducted to evaluate the efficacy and safety of viscum fraxini-2 in patients with chemotherapy–naïve, advanced hepatocellular carcinoma. 23 patients with unrespectable HCC who had received no prior systemic chemotherapy with objectively measurable tumors were enrolled on this study. The mistletoe preparation for the study is an aqueous injectable solution. It contains one milliliter of viscum fraxini in dilution stage–2 (15 mg extract of 20 mg mistletoe herb from ash tree, diluted in di-natrium-mono-hydrogen phosphate, ascorbic acid and water) which is equivalent to 10 000 ng/ml injection ampoules. 2 ampoules of viscum fraxini–2 were administered subcutaneously once weekly. As assessed by conventional imaging criteria, 3 (13.1%) patients have achieved complete response, 2 (8.1%) patients have achieved a partial response. 9 (39.1%) had progressive disease while 9 (39.1%) patients didn't have evaluation of response due to early death. The median overall survival time for all patients was 5 months (range 2–38 months), for those who achieved a CR was 29 months (range 12–38 months) and, for those who achieved a PR was 6.5 months (range 6–7 months). The median progression free survival for all patients was 2 months (range 1–38 months), for those who achieved a CR, it was 29 months (range 8–38 months) and for those who achieved a partial response, it was 5 months (range 4–6 months). No hematologic toxicity has been encountered. The spectrum of non-hematologic toxicity was mild. The WHO toxicity criteria grade 3–4 were 34.8% drug related fever, 13.1% erthyma at injection site and 17.4% pain at the site of injection. No drug related discontinuation or toxic deaths have occurred. Viscum fraxini-2 seems to be particularly promising in patients with advanced HCC, it shows antitumor activity and low toxicity profile. Further studies in combination with other active agents are clearly warranted.

Hepatocellular carcinoma (HCC) is one of the most common cancers worldwide. Although it is far less common in western countries, it is the most common malignant tumor in areas of Africa and Asia (Bosch and Munoz, 1991; Lotze *et al*, 1993). Although a wide range of therapeutic options is available, the efficacy of these methods and the prognosis of patients with HCC remain very poor. Surgical resection represents the only possibility of cure. However, resection rates for patients with HCC remain low because of a high incidence of associated cirrhosis, the direct invasion of the tumor into the portal or the hepatic veins, or early spread to the entire liver. Many non-surgical local treatments, such as cryosurgery and radiation therapy, have been proposed; however, considerable uncertainty remains about their effectiveness (Venook, 1994). Eventually, in most patients with HCC, the disease progress to a far-advanced stage for which effective local treatment is not available. These findings stress the pressing need for efficacious, systemic chemotherapy for patients with inoperable HCC.

The role of chemotherapy in the treatment of patients with HCC remains controversial. Numerous single chemotherapeutic agents and drug combinations have been given to HCC patients in an attempt to alter their predictably short survival time. Unfortunately, the activity of a single agent is limited, with only a few drugs showing a response rate >10%. Moreover, combination chemotherapy has proven equally disappointing, because additional drugs have resulted in increased toxicity without any increased efficacy compared with single–agent doxorubicin therapy (Whang-Peng and Chao, 1998). Therefore, there is no drug or protocol of treatment that can be recommended as standard therapy for this group of patients. Due to the lack of any effective systemic chemotherapy, there is an urgent need to investigate new drugs.

Viscum album L. is a semi parasitic plant growing on different host trees (Becker, 1986). The extracted mistletoes are composed of many biologically active substances. The principle of the mistletoe phytotherapeutics can be considered as combined cytotoxic and biological response modifying activities that result from the activities of the plant lectins and other biologically relevant substances (Zarkovic *et al*, 2001). In 1920, Steiner recommended the mistletoe as a remedy against cancer (Steiner, 1961). In a systematic review on controlled clinical trials, twenty-three studies were identified: 16 randomized, 2 quasi-randomized and 5 non-randomized. Cancer sites included breast, lung, stomach, colon, rectum, head and neck, kidney, bladder, melanoma, glioma, and genital. Among these studies, statistically significant positive outcomes were reported for survival (*n*=8), tumor remission (*n*=1), overall quality of life (*n*=3), and quality of life in relation to side effects during cytoreductive therapy (*n*=3) (Kienle *et al*, 2003).

Viscum Fraxini is an aqueous extract of mistletoe (Viscum album L. grown on ash trees) (Koehler, 1992) and it is the preparation with the highest lectin content (Scheer, 1996). We herein, report the results of a phase II study to evaluate its efficacy and safety in the treatment of patients with advanced HCC.

## PATIENTS AND METHODS

### Eligibility criteria

The eligibility criteria included: (1) pathology proven primary HCC or *α*-fetoprotein >400 ng/ml with a hepatic tumor highly suggestive of HCC by imaging studies; (2) unresectable tumor and patient was not a candidate for either transcatheter arterial chemoembolization (TACE) or percutaneous ethanol injection (PEI); (3) bi-dimensional measurable disease; (4) no previous systemic chemotherapy; (5) age between 16 years and 75 years; (6) performance status 0–3 WHO, 7) within normal renal, cardiac and hematological profile.

### Treatment protocol

Prior to entry into the study, all patients provided a complete history and physical examination, including performance status, concurrent nonmalignant diseases and therapy. Laboratory studies included a complete blood cell counts, differential count, biochemical liver and renal function tests, electrolyte, chest x-rays, *α*-fetoprotein, triphasic liver computed scan (CT) and Child class evaluation were performed before treatment. The mistletoe preparation for the study is an aqueous injectable solution. It contains one milliliter of viscum fraxini in dilution stage–2 (15 mg extract of 20 mg mistletoe herb from ash tree, diluted in di-natrium-mono-hydrogen phosphate, ascorbic acid and water) which is equivalent to 10 000 ng/ml injection ampoules. 2 ampoules of viscum fraxini–2 were administered subcutaneously once weekly. Patients were seen on a weekly basis during treatment for history taking and physical examination. A complete blood count was determined every week. Renal and liver functions and *α*-fetoprotein levels were examined every 4 weeks. The tumor was assessed by CT every 8 weeks.

### Definition of response

Determination of the tumor response followed standard response criteria established by the World Health Organization (WHO) (Miller *et al*, 1981). Complete response (CR) was defined as the complete disappearance of all known lesions on radiological grounds for at least 4 weeks. Partial response (PR) was defined as a decrease of 50% or more in the product of two perpendicular diameters of the largest tumor nodule for at least 4 weeks without the appearance of new lesions or progression of lesions. Static disease (SD) was defined as a <50% decrease, or not more than a 25% increase, in the product of two perpendicular diameters of the largest tumor nodule. Progressive disease (PD) was defined as >25% increase in the product of two perpendicular diameters of the largest tumor nodule or one of the measurable lesions, or the appearance of new lesions. Patients who did not survive to reassessment by radiological methods were considered to have undetermined response (UR).

### Toxicity

Evaluation of toxicity, classified according to the criteria of the WHO (World Health Organization, 1979) included physical examination prior to every injection, complete blood cell counts and serum assessment of renal and hepatic functions. Each patient after having at least one dose of protocol therapy was evaluable for toxicity.

### Statistical analysis

Descriptive statistics are reported as percentages and medians. The overall survival time was calculated from the start of therapy to the date of death or the last visit of the patient. The time to disease progression was defined from the start of therapy to the date of disease progression. Survival curves were constructed using the Kaplan-Meier productlimit method (Kaplan and Meier, 1959).

### Study approval

The study was approved by the local ethics committee and all patients signed informed consent before entering the study.

## RESULTS

### Patient characteristics

Base line patient characteristics and clinical features are summarized in Table 1

**Table 1:** Patients Characteristics

**Characteristic**	**No. of patients**	%
Male: female (total)	20 : 3 (23)	
Median age in years (range)	54 (39–75)	
		
*Performance status*		
1	10	43.5
2	7	30.4
3	6	26.1
		
*Hepatitis*		
HbsAg (+)	13	56.5
Anti HCV (+)	8	34.8
Both (+)	2	8.7

*Child's classification*		
A	9	39.1
B	6	26.1
C	8	34.8
*Okuda stage:*		
I	4	17.4
II	12	52.2
III	7	30.4

*Diagnosis:*		
Cytology	14	60.9
*α*-fetoprotein and imaging	9	39.1

*Tumor status*		
Bilateral lobe affection	8	34.8
Unilateral lobe affection	15	65.2
Distant metastasis	4	17.4
Main Portal vein Thrombosis	2	8.7
Ascites	4	17.4

. Twenty-three patients were entered into the trial. They were 20 males and 3 females, aged 39–75 years with a median of 54 years. Their WHO performance status was 1 in 10 patients (43.5%), 2 in 7 patients (30.4%) and 3 in 6 patients (26.1%). HbsAg and anti HCV were positive in 13 patients (56.5%) and 8 patients (34.8%), respectively, while 2 patients (8.7%) were positive for both. All patients were chemotherapy naı"ve. The median duration of treatment on viscum fraxini-2 is 17 weeks (range 3–152 weeks). Okuda stage I was found in 4 patients (17.4%), stage II in 12 patients (52.2%) and stage III in 7 patients (30.4%). The diagnosis of HCC was based on fine-needle aspiration cytology of liver tumors in 14 patients (60.9%). The remaining 9 patients (39.1%) were diagnosed by marked elevation of *α*-fetoprotein level and imaging studies indicating advanced HCC. All of the patients had far-advanced HCC at the time of diagnosis. Bilateral lobar affection of the liver was present in 8 patients (34.8%). Extensive one lobe affection was present in 15 patients (65.2%). Distant metastasis were present in 4 patients (17.4%) (2 bone metastasis and 2 lymph node metastasis). Main portal vein thrombosis was detected in two patients (8.7%) while ascites was present in 4 patients (17.4%).

### Response

**Figure 1:**
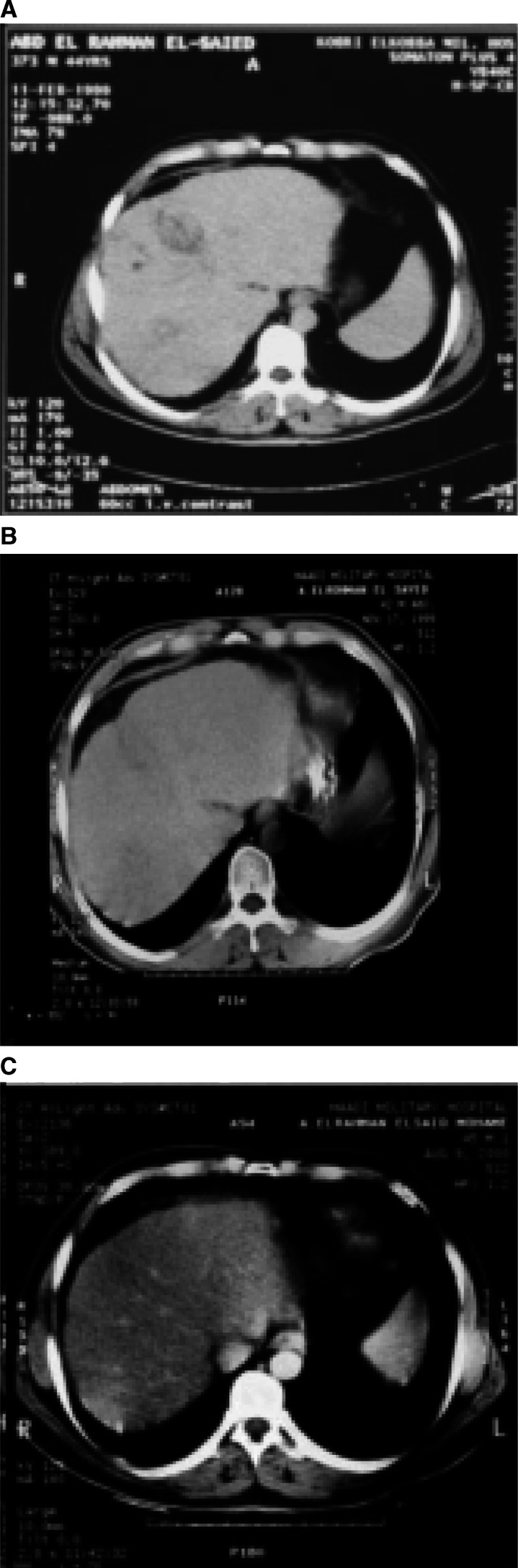
A case of hepatocellular carcinoma with multiple focal lesions before treatment (**A**) with regression of the tumor (**B**) and disappearance of the tumor (**C**) after 6 months of viscum fraxini-2 therapy.

According to conventional radiological response criteria, 3 patients (13.1%) achieved complete response. The first patient achieved CR after 4 months from starting the treatment and remained disease free for 4 months. The second and the third patients achieved CR after 6 months and they are still living disease free for more than 29 and 38 months, respectively (CT scan of the third patients before treatment and after disappearance of the tumor is shown in Figure 1

). 2 patients (8.7%) achieved partial 9 response. Progressive disease has been shown in 9 patients (39.1%). 9 patients (39.1%) did not have evaluation of response due to early death and they were classified as UR.

### Survival

**Figure 2:**
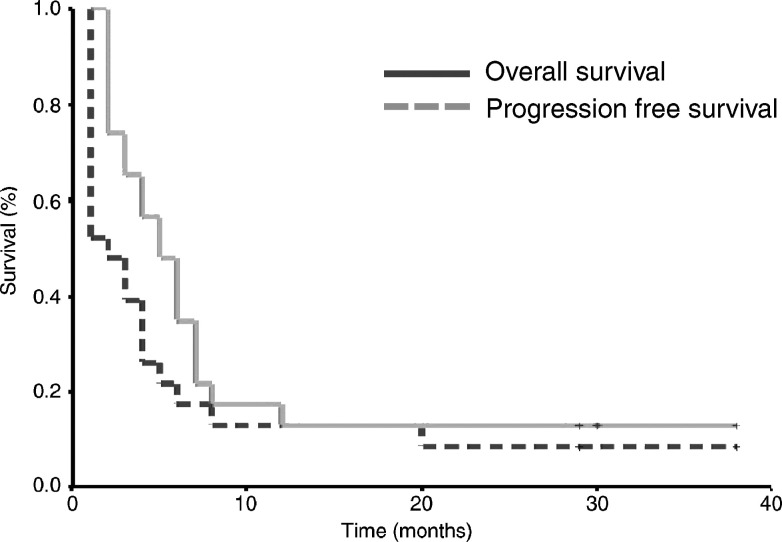
Acturial overall and progression free survival curve.

At the time of analysis, 3 (13.1%) patients remained alive including two patients with CR and one patient with slowly progressive disease. The median overall survival time for all patients was 5 months (range 2–38 months), for those who achieved a CR was 29 months (range 12–38 months) and, for those who achieved a PR was 6.5 months (range 6–7 months). The median progression free survival for all patients was 2 months (range 1–38 months), for those who achieved a CR, it was 29 months (range 8–38 months) and for those who achieved a partial response, it was 5 months (range 4–6 months). The Kaplan- Meier actuarial overall survival and PFS curves for all patients are shown in Figure 2

.

### Toxicity

All patients were evaluated for toxicity. Drug related fever developed in 8 patients (34.8%). Erythma at injection site developed in 3 patients (13.1%). 4 patients (17.4%) suffered pain at site of injection. 3 patients had to reduce the dose to one ampoule on the subsequent treatment courses. Anti-inflammatory and analgesics were used in only one patient due to severe pain and erythma at injection site. There were no drug related discontinuation or toxic deaths.

## DISCUSSION

Hepatocellular carcinoma is one of the most common cancers worldwide (Okuda, 1980). Resection is perhaps the only meaningful chance of cure. Although early diagnosis is becoming more frequent, particularly in those populations subjected to screening programs, resection rates for patients with HCC remain low (Lee *et al*, 1982 and Okuda *et al*, 1985). Therefore, the vast majority of patients with HCC are not candidates for curative surgery or other local therapy and systemic chemotherapy may be the only option for their treatment.

The antitumor activities of a number of chemotherapeutic agents have been evaluated in HCC patients, but most yielded poor results and probably are associated with severe side effects. Doxorubicin remains the most active drug against HCC, with a single agent tumor response rate of about 10–20%. However, its toxicity outweighs its benefit (Lai *et al*, 1988; Lai *et al*, 1990). Chemotherapeutic agents other than doxorubicin have demonstrated even less activity and progress in treating HCC patients with chemotherapy has been disappointing. New chemotherapeutic drugs, such as paclitaxel, raltrexed, irinotecan, and nolatrexed, have not demonstrated encouraging results. These new drugs exhibit some antitumor activity, but response rates rarely exceed 10% (Rougier *et al*, 1997; Chao *et al*, 1998; O'Reilly *et al*, 1998; Stuart *et al*, 1999). The possible explanations for the refractioness of HCC to chemotherapy include tumor heterogeneity (Dexter and Leith, 1986), inadequate dosage of anticancer agents (Lai *et al*, 1990) and the inducible overexpression of the multidrug resistance gene (Huang *et al*, 1992). Therefore, all patients with advanced HCC should be considered for joining well– designed phase II trials with novel antitumor agents or regimens when patients can tolerate treatment.

Viscum fraxini is an aqueous extract of mistletoe. Controversy exists as to how the plant extracts exert their postulated dual activity of cytotoxicity towards tumor cells and stimulation of immune cells (Janssen *et al*, 1993).

Immunostimulatory effects of mistletoe extract have been assigned to a low molecular weight oligosaccharide compound (Hamprecht *et al*, 1987; Mueller *et al*, 1989). Moreover, the stimulatory effect on natural killer (NK) cell activity appears to result from enhanced production of interferon (INF-*γ*) and tumor necrosis factor-*α* (TNF-*α*) by T cells and macrophages respectively (Mueller and Anderer, 1990). On the other hand, there is some evidence that lectin components are important effector molecules in mistletoe preparations (Luther and Becker, 1987). The lectin components increase the total number and the activities of neutrophils, NK cells and large granular lymphocytes as well as they activate simultaneously monocyte-macrophage and helper cell subpopulations. This was associated with higher levels of cytokines such as interleukins IL-1, IL-6, granulocyte macrophage colony stimulating factor (GM-CSF) and TNF-*α* (Hajto *et al*, 1990, Schultze *et al*, 1991; Beuth *et al*, 1993).The described immunological effects of mistletoe polysaccharides are supplemented by membrane lipids. As shown by Hartmann *et al* (1995) isolated vesicles of genuine membrane systems were the most potent stimulating factor for T-cell proliferation of mistletoe extracts (Hartmann *et al*, 1995).

Apart from the immunostimulatory effect of mistletoe extract and lectins, it has been reported that they have a direct cytotoxic effect (Ribereau–Gayon *et al*, 1986; Urech *et al*, 1996). Lectins are considered essential components of mistletoe aqueous extract responsible for apoptosis (Janssen *et al*, 1993). Viscotoxins which are a group of cytotoxic polypeptides belonging to the class of thionins (Schrader-Fischer and Apel, 1993) can enhance the permeability of cell membrane (Lankish and Vogt, 1978). So, mistletoe extracts are able to induce both ways of biological cell death; apoptosis and lysis. Moreover, these initial effects subsequently induce immune stimulating reactions.

Taken together, complete mistletoe extract containing membrane lipids like viscum fraxini affects both arms of immune system, the T-cell and the B-cell parts, initially inducing proliferation and activation of immune competent cells as well as expression of cytokines, supporting antitumoral effects.

In the light of these observations, the present study aimed to improve efficacy of systemic chemotherapy. HCC patients with inoperable disease or exrtahepatic disease who were not suitable for regional intra-arterial treatment were enrolled in this study. The treatment resulted in an overall response rate of 21.74% (5/23) with a good impact on survival. While most of Phase II trials of treatment of HCC confirmed that there is no effective drug for HCC and all single systemic anticancer agents produced a response rate of less than 10% (Yoshida *et al*, 1988; Lin *et al*, 1993; Okada *et al*, 1993; Wierzbicki *et al*, 1994; Falkson and Burger, 1995), Viscum Fraxini-2 in our study showed encouraging results and might represent a good opportunity for treating patients with advanced HCC.

In conclusion, Viscum Fraxini-2 is active in HCC with anti-tumor activity and low toxicity profile. Further studies in combination with other active agents are clearly warranted.
